# Functional *AGXT2* SNP rs180749 variant and depressive symptoms: Baseline data from the Aidai Cohort Study in Japan

**DOI:** 10.1007/s00702-024-02742-w

**Published:** 2024-01-23

**Authors:** Hiroshi Kumon, Yoshihiro Miyake, Yuta Yoshino, Jun-ichi Iga, Keiko Tanaka, Hidenori Senba, Eizen Kimura, Takashi Higaki, Bunzo Matsuura, Ryuichi Kawamoto, Shu-ichi Ueno

**Affiliations:** 1 Department of Neuropsychiatry, Molecules and Function, Ehime University Graduate School of Medicine, Ehime, 791-0295 Japan; 2 Department of Epidemiology and Public Health, Ehime University Graduate School of Medicine, Ehime, Japan; 3 Integrated Medical and Agricultural School of Public Health, Ehime University, Ehime, Japan; 4 Research Promotion Unit, Translation Research Center, Ehime University Hospital, Ehime, Japan; 5 Center for Data Science, Ehime University, Ehime, Japan; 6 Department of Internal Medicine, Matsuyama Shimin Hospital, Ehime, Japan; 7 Department of Medical Informatics, Ehime University Graduate School of Medicine, Ehime, Japan; 8 Department of Regional Pediatrics and Perinatology, Ehime University Graduate School of Medicine, Ehime, Japan; 9 Department of Lifestyle-Related Medicine and Endocrinology, Ehime University Graduate School of Medicine, Ehime, Japan; 10 Department of Community Medicine, Ehime University Graduate School of Medicine, Ehime, Japan

**Keywords:** AGXT2, Depressive symptoms, Japanese, Functional single nucleotide polymorphisms, CES-D

## Abstract

No study has shown the relationship between alanine-glyoxylate aminotransferase 2 (*AGXT2*) single nucleotide polymorphisms (SNPs) and depressive symptoms. The present case–control study examined this relationship in Japanese adults. Cases and control participants were selected from those who participated in the baseline survey of the Aidai Cohort Study, which is an ongoing cohort study. Cases comprised 280 participants with depressive symptoms based on a Center for Epidemiologic Studies Depression Scale (CES-D) score ≥ 16. Control participants comprised 2034 participants without depressive symptoms based on the CES-D who had not been diagnosed by a physician as having depression or who had not been currently taking medication for depression. Adjustment was made for age, sex, smoking status, alcohol consumption, leisure time physical activity, education, body mass index, hypertension, dyslipidemia, and diabetes mellitus. Compared with the GG genotype of rs180749, both the GA and AA genotypes were significantly positively associated with the risk of depressive symptoms assessed by the CES-D: the adjusted odds ratios for the GA and AA genotypes were 2.83 (95% confidence interval [CI] 1.23–8.24) and 3.10 (95% CI 1.37–8.92), respectively. The TGC haplotype of rs37370, rs180749, and rs16899974 was significantly inversely related to depressive symptoms (crude OR 0.67; 95% CI 0.49–0.90), whereas the TAC haplotype was significantly positively associated with depressive symptoms (crude OR 1.24; 95% CI 1.01–1.52). This is the first study to show significant associations between *AGXT2* SNP rs180749, the TGC haplotype, and the TAC haplotype and depressive symptoms.

## Introduction

Major depressive disorder (MDD) is a common psychiatric disorder. Environmental and genetic factors are interrelated in the development of MDD. Environmental factors such as emotional abuse and neglect were found to be related to the risk for depressive symptoms (Mandelli et al. 2015; Kwong et al. 2019). As for genetic factors, 102 independent variants and 269 genes were identified in a genome-wide meta-analysis of MDD (Howard et al. 2019). Moreover, recent findings have indicated that epigenetics, including DNA methylation, non-coding RNAs, and histone modifications, are related to the pathogenesis of MDD (Penner-Goeke and Binder 2019; Ménard et al. 2016).

Alanine-glyoxylate aminotransferase 2 (AGXT2) was first identified as an important enzyme related to the metabolism of R-3-amino isobutyrate in 1969 (Kakimoto et al. 1969b). *AGXT2* single-nucleotide polymorphisms (SNPs) are related to systemic diseases [coronary artery disease (Amir et al. 2018), carotid atherosclerosis (Yoshino et al. 2014b), chronic heart failure (Hu et al. 2016), diabetes mellitus (DM) (Kumon et al. 2022), and mild cognitive impairment (Granot-Hershkovitz et al. 2023)] and biological parameters [blood pressure, sugar, and creatinine (Yoshino et al. 2021)]. Missense *AGXT2* SNPs rs37370 (S102N), rs37369 (V140I), rs180749 (T212I), and rs16899974 (V498L) and the haplotype of those SNPs were found to be associated with loss of AGXT2 activity (Yoshino et al. 2014a, 2021). Lack of AGXT2 activity is shown in 30–40% of Japanese persons, compared to less than 10% of Caucasian individuals (Kakimoto et al. 1969a, b).

AGXT2 also regulates methylarginines such as asymmetric *N*^G^, *N*^G^-dimethyl-l-arginine (ADMA) and symmetric *N*^G^, *N*′^G^-dimethyl-l-arginine (SDMA) (Ogawa et al. 1987). ADMA is an endogenous inhibitor of nitric oxide synthase (NOS). ADMA dose-dependently inhibits endothelial NOS (eNOS) and neuronal NOS (nNOS) activity in the presence of physiologic cellular l-arginine (Kielstein et al. 2007). AGXT2 impacts the nitric oxide (NO) pathway through the competitive inhibition of NOS by ADMA; as a result, AGXT2 can regulate blood pressure and be related to endothelial dysfunction leading to cardiovascular disease (Yoshino et al. 2021; Rochette et al. 2013). In MDD patients, elevated ADMA and decreased SDMA concentrations were reported (Baranyi et al. 2015). Moreover, decreased peripheral production of NO and plasma concentration of NO were found in MDD patients (Chrapko et al. 2006; Selley 2004). Because NO modulates release of neurotransmitters such as acetylcholine, catecholamines, serotonin, and histamine, reduction of NO in the brain may be related to the pathogenesis of MDD (Prast and Philippu 2001).

The Center for Epidemiologic Studies Depression Scale (CES-D), a short self-report for evaluating depressive symptomatology in the general population, has been widely used to measure depressive symptoms. In a meta-analysis of 28 studies, CES-D had acceptable screening accuracy in the general population (Vilagut et al. 2016) and was useful for adolescents and older adults (Blodgett et al. 2021; Cosco et al. 2020). The present case–control study was conducted to investigate the relationship between four functional *AGXT2* SNPs and depressive symptoms based on the CES-D in Japanese adults using baseline data from the Aidai Cohort Study (AICOS).

## Materials and methods

### Study population

The AICOS is an ongoing, population-based, prospective study that was started in 2015 (Fukui et al. 2019; Miyake et al. 2020, 2021, 2023; Hara et al. 2020; Tanaka et al. 2021; Nobori et al. 2022; Kumon et al. 2022). The baseline survey of the AICOS was conducted in Yawatahama City in 2015, Uchiko Town in 2016, Seiyo City in 2017, and Ainan Town in 2017, with total populations of approximately 36,000, 17,000, 38,000, and 22,000, respectively; these four municipalities are among the 20 municipalities in Ehime Prefecture on Shikoku Island, situated south of Japan’s main island. Study participants were recruited from individuals who had undergone health checkups conducted by their municipality of residence or through alternative recruitment procedures, and 798, 347, 524, and 755 study participants in Yawatahama City, Uchiko Town, Seiyo City, and Ainan Town, respectively, took part in the baseline survey of the AICOS. In addition, 223 employees of a manufacturing company (PHC Corporation) in Toon City, one of the municipalities in Ehime Prefecture, also participated in the AICOS. In total, 2,647 participants aged 33–89 years (1069 men aged 35–89 years and 1578 women aged 33–85 years) gave written, informed consent and completed a self-administered questionnaire; however, 268 of them had missing or incomplete data for the factors under study and were excluded from the present study. A Japanese version (Shima 1985) of the CES-D, a 20-item questionnaire (Radloff 1977), was incorporated into the questionnaire. Study subjects were asked to score the frequency of the occurrence of specific symptoms over the last week on a 4-point scale (0, “less than 1 day”; 1, “1–2 days”; 2, “3–4 days”; and 3, “5–7 days”). All sub-scores were summed to yield a total score ranging from 0 to 60, and the presence of depressive symptoms was defined as a CES-D score ≥ 16 (Radloff 1977; Shima 1985). In the current case–control study, 280 cases of depressive symptoms based on a CES-D score ≥ 16 were identified. Of the 2099 remaining study participants who were eligible to serve as controls, 65 who were not considered to have depressive symptoms as defined by the CES-D criteria but who had been diagnosed by a physician as having depression or who had been currently taking medication for depression were excluded. The final analyses thus included 280 cases and 2034 control participants. The AICOS was approved by the ethics committee of the Ehime University Graduate School of Medicine (approval No. 1504012).

### Measurements

Information on age, sex, smoking habits, alcohol drinking habits, physical activity, education, and current use of antihypertensive, cholesterol-lowering, and diabetic medications was also elicited using the questionnaire at baseline. Research technicians completed missing or illogical data by telephone interview. Never smoking was defined as having smoked fewer than 100 cigarettes in their lifetime. Former smoking was defined as having smoked more than 100 cigarettes in their lifetime but having quit smoking by the time of the survey. Current smoking was defined as having smoked more than 100 cigarettes in their lifetime and continuing to smoke at the time of the baseline survey. Leisure time physical activity was defined as at least 30 min of any type of moderate-to-vigorous physical activity, such as brisk walking, golf, gardening, jogging, or playing tennis, at least once a week. Body height and weight were measured in light clothing without shoes, and the body mass index (BMI) was computed by dividing body weight in kilograms by the square of the height in meters. Blood pressure was measured twice, each time with the study participant in the sitting position after at least 5 min of rest, using an automated sphygmomanometer. The second measurement was used for the present study. Hypertension was defined as current use of antihypertensive medication, systolic blood pressure ≥ 140 mmHg, or diastolic blood pressure ≥ 90 mmHg. After overnight fasting, blood samples were collected from an antecubital vein. Plasma glucose concentrations, hemoglobin A1c levels, serum low-density cholesterol, high-density lipoprotein cholesterol, and triglyceride concentrations were measured at an external laboratory (Shikoku Chuken, Ehime, Japan). Dyslipidemia was defined as current use of cholesterol-lowering medication, serum low-density lipoprotein cholesterol concentration ≥ 140 mg/dL, high-density lipoprotein cholesterol concentration < 40 mg/dL, or triglyceride concentration ≥ 150 mg/dL. DM was defined as current use of diabetic medication, fasting plasma glucose level ≥ 126 mg/dL, or hemoglobin A1c level ≥ 6.5%.

### DNA extraction and genotyping

After 7 mL of blood samples treated with EDTA were stored at 2–5 °C within 3 days, blood samples were centrifuged at room temperature and the buffy coat was collected. Genomic DNA was prepared from the buffy coat using a DNA extraction kit (QuickGene-610L; FUJIFILM Wako Pure Chemical Corp., Osaka, Japan) and stored at − 20 °C. According to the manufacturer’s instructions, four *AGXT2* SNPs, rs37370 (C__1018750_1_), rs37369 (C__11162986_1_), rs180749 (C__1018735_1_), and rs16899974 (C__25742181_10), were genotyped using the prestandardized and experimentally validated TaqMan SNP genotyping assay on the StepOnePlus machine (Applied Biosystems, Foster City, CA, USA).

### Statistical analysis

SNPs under study were tested for deviation from Hardy–Weinberg equilibrium using the Chi-squared test. Linkage disequilibrium analysis was performed using Haploview software version 4.2 (Broad Institute, Cambridge, MA) (Barrett et al. 2005). Logistic regression analysis was performed to estimate crude odds ratios (ORs) and 95% confidence intervals (CIs) for depressive symptoms relative to the SNPs under investigation, with the reference category being the homozygote of the ancestral allele based on the National Center for Biotechnology Information SNP database. Multiple logistic regression analysis was performed to adjust for age, sex, smoking status, alcohol consumption, leisure time physical activity, education, BMI, hypertension, dyslipidemia, and DM. Haplotypes and their frequencies were inferred by means of the expectation maximization algorithm using STATA/MP software version 17.0 (StataCorp, College Station, TX, USA). For differences in haplotype frequencies between the cases and controls, crude ORs and 95% CIs were estimated based on the frequency of each haplotype relative to all other haplotypes combined. Except for the calculation of linkage disequilibrium and haplotype analysis, all analyses were conducted using the SAS software package version 9.4 (SAS Institute, Inc., Cary, NC, USA).

## Results

### Comparison of cases with control subjects

Compared with the control participants, cases were more likely to be younger and female and were less likely to report current alcohol consumption and leisure time physical activity (Table 1). No differences were found between cases and controls with respect to smoking status, education, BMI, hypertension, dyslipidemia, and DM.

**Table 1:** Characteristics of the study population

Variable	n (%) or mean ± SD		
	Cases (N = 280)	Controls (N = 2034)	*P* value^a^
Age, years, mean ± SD	58.5 ± 11.0	61.4 ± 10.4	** < 0.0001**
Male sex, n (%)	84 (30.0)	862 (42.4)	** < 0.0001**
Smoking status, n (%)			0.79
Never	189 (67.5)	1,348 (66.3)	
Former	66 (23.6)	516 (25.4)	
Current	25 (8.9)	170 (8.4)	
Alcohol consumption, n (%)			**0.046**
Never	126 (45.0)	824 (40.5)	
Former	25 (8.9)	128 (6.3)	
Current	129 (46.1)	1,082 (53.2)	
Leisure time physical activity, n (%)	89 (31.8)	904 (44.4)	** < 0.0001**
Education, years, n (%)			0.50
< 13	31 (11.1)	271 (13.3)	
13–14	139 (49.6)	952 (46.8)	
≥ 15	110 (39.3)	811 (39.9)	
Body mass index, kg/m^2^, mean ± SD	23.4 ± 3.9	23.4 ± 3.3	0.34
Hypertension, n (%)	110 (39.3)	911 (44.8)	0.08
Dyslipidemia, n (%)	161 (57.5)	1088 (53.5)	0.21
Diabetes mellitus, n (%)	16 (5.7)	171 (8.4)	0.12

*SD* standard deviation

^a^Chi-squared test or Wilcoxon rank-sum test

Two-sided *P* values less than 0.05 were considered statistically significant.

### Characteristics of *AGXT2* polymorphisms

Among the control participants, *AGXT2* SNPs rs37370, rs180749, and rs16899974 did not deviate from Hardy–Weinberg equilibrium (*P* = 0.18, 0.29, and 0.13, respectively) (Table 2A). On the other hand, *AGXT2* SNP rs37369 deviated (*P* = 0.01); therefore, *AGXT2* SNP rs37369 was excluded from the present study. *AGXT2* SNPs rs37370, rs180749, and rs16899974 showed weak linkage disequilibrium (Table 2B).

**Table 2:** Select tagging *AGXT2* SNPs and pairwise linkage disequilibrium of *AGXT2* polymorphisms

(A) Main characteristics of the select tagging *AGXT2* SNPs in control subjects				
*AGXT2* SNP (major/minor allele)	Localization	HWE p value	MAF	Genotyping rate (%)
rs37370 (T/C)	Exon 3 (S102N)	0.18	G (0.45)	99.8
rs37369 (T/C)	Exon 4 (V140I)	**0.01**	C (0.36)	100
rs180749 (A/G)	Exon 6 (T212I)	0.29	G (0.21)	100
rs16899974 (C/A)	Exon 14 (V498L)	0.13	A (0.47)	99.95

(B) Pairwise linkage disequilibrium of *AGXT2* polymorphisms (*r*^*2*^ below and D′ above the diagonal)			
	rs37370	rs180749	rs16899974
rs37370		0.79	0.29
rs180749	0.13		0.37
rs16899974	0.08	0.03	

*MAF* minor allele frequency

Two-sided *P* values less than 0.05 were considered statistically significant.

### Relationship between *AGXT2* polymorphisms and depressive symptoms

Compared with study participants with the GG genotype of *AGXT2* SNP rs180749, those with the GA and AA genotypes had a significantly increased risk of depressive symptoms (Table 3). After adjustment for confounders under study, the positive associations were slightly strengthened: the adjusted ORs for the GA and AA genotypes were 2.83 (95% CI 1.23–8.24) and 3.10 (95% CI 1.37–8.92), respectively, with rs37370 and rs16899974 not materially associated with the risk of depressive symptoms in the multivariate model.

**Table 3:** ORs and 95% CIs for depressive symptoms in relation to *AGXT2* polymorphisms

SNP^a^	Genotype	Cases *n* (%)	Controls *n* (%)	Crude OR (95% CI)	Adjusted OR (95% CI)^b^
rs37370		(*N* = 280)	(*N* = 2029)		
	CC	52 (18.6)	419 (20.7)	1.00	1.00
	CT	133 (47.5)	972 (47.9)	1.10 (0.79–1.56)	1.08 (0.77–1.54)
	TT	95 (33.9)	638 (31.4)	1.20 (0.84–1.73)	1.20 (0.84–1.74)
rs180749		(*N* = 280)	(*N* = 2034)		
	GG	5 (1.8)	100 (4.9)	1.00	1.00
	GA	89 (31.8)	664 (32.7)	**2.68 (1.17**–**7.75)**	**2.83 (1.23**–**8.24)**
	AA	186 (66.4)	1270 (62.4)	**2.93 (1.30**–**8.38)**	**3.10 (1.37**–**8.92)**
rs16899974		(*N* = 280)	(*N* = 2033)		
	CC	71 (25.4)	561 (27.6)	1.00	1.00
	CA	144 (51.4)	1047 (51.5)	1.09 (0.81–1.48)	1.10 (0.81–1.50)
	AA	65 (23.2)	425 (20.9)	1.21 (0.84–1.73)	1.28 (0.89–1.85)

^a^*AGXT2* SNPs, rs37370 (C__1018750_1_), rs180749 (C__1018735_1_), and rs16899974 (C__25742181_10), were genotyped using the prestandardized and experimentally validated TaqMan SNP genotyping assay.

^b^Adjusted for age, sex, smoking status, alcohol consumption, leisure time physical activity, education, body mass index, hypertension, dyslipidemia, and diabetes mellitus.

Bold text indicates statistical significance

*CES-D* Center for Epidemiologic Studies Depression Scale, *CI* confidence interval, *OR* odds ratio

### Haplotype analysis of three *AGXT2* polymorphisms in relation to depressive symptoms

In both cases and controls, seven haplotypes with a frequency of 1% or more were detected (Table 4). Given that the haplotype order was rs37370, rs180749, and rs16899974, the TGC haplotype was significantly related to a reduced risk of depressive symptoms in comparison with all other haplotypes combined: the crude OR was 0.67 (95% CI 0.49–0.90). In contrast, the TAC haplotype was significantly positively associated with the risk of depressive symptoms compared with all other haplotypes combined: the crude OR was 1.24 (95% CI 1.01–1.52).

**Table 4:** Haplotype analysis of three *AGXT2* polymorphisms in relation to depressive symptoms

Haplotype^a^	Frequency n (%)		Crude OR (95% CI)^b^
	Cases (2*N* = 560)	Controls (2*N* = 4068)	
CGC	9 (1.6)	66 (1.6)	0.99 (0.43–2.01)
CAC	73 (13.0)	621 (15.3)	0.83 (0.63–1.08)
CAA	155 (27.7)	1117 (27.5)	1.01 (0.82–1.24)
TGC	53 (9.5)	550 (13.5)	**0.67 (0.49**–**0.90)**
TGA	37 (6.6)	232 (5.7)	1.17 (0.79–1.68)
TAC	151 (27.0)	934 (23.0)	**1.24 (1.01**–**1.52)**
TAA	82 (14.6)	532 (13.1)	1.14 (0.88–1.47)

Rare haplotypes (frequency less than 1% in either cases or controls) were deleted. Bold text indicates statistical significance

*CI* confidence interval, *OR* odds ratio

^a^Haplotype order is rs37370, rs180749, and rs16899974

^b^Crude OR for each haplotype is relative to all other haplotypes combined

## Discussion

To the best of our knowledge, the current case–control study is the first to investigate the relationship between *AGXT2* SNPs (rs37370, rs180749, and rs16899974) and depressive symptoms. The present study showed that the GA and AA genotypes of rs180749 were significantly positively associated with the risk of depressive symptoms compared with the GG genotype. No relationships were found between *AGXT2* SNPs rs37370 or rs16899974 and depressive symptoms. Haplotype analyses identified a significant inverse association between the TGC haplotype of rs37370, rs180749, and rs16899974, occurring in 13.5% of the controls, and depressive symptoms and a significant positive relationship between the TAC haplotype, occurring in 23.0% of the controls, and depressive symptoms.

The GA and AA genotypes of rs180749 are related to loss of AGXT2 activity (Yoshino et al. 2014a) and can cause ADMA metabolic attenuation. Elevated plasma ADMA levels and decreased circulating NO levels were shown in Agxt2-knockout mice (Caplin et al. 2012). Overexpression of Agxt2 in vitro conversely increased NO production and protected endothelial cells from ADMA-induced inhibition of NO production (Rodionov et al. 2010). In fact, decreased cerebral blood flow in the right anterior cingulate and frontal gyri was shown in persons with MDD (Monkul et al. 2012). Moreover, cerebrovascular disease identified as white matter hyperintensities on T2-weighted MRI was also related to depression, especially late-life depression, and cognitive dysfunction (Taylor et al. 2013). Because ADMA impacts to the regulation of blood pressure, AGXT2 might be related to depressive symptoms through the change of ADMA metabolism that can modify cerebral blood flow (Kielstein et al. 2006).

Although AGXT2 haplotypes also may impact to depressive symptoms via the ADMA metabolism, there is no report on how AGXT2 haplotypes of rs37370, rs180749, and rs16899974 affects the AGXT2 function and the ADMA metabolism.

Two methodological strengths of the current study are that the study participants were homogeneous with respect to their residential area and that adjustment was made for several confounders.

There were several limitations of the present study. The participants were probably not representative of the Japanese general population because the participation rate was low in the baseline survey of the AICOS. For example, the educational level of the present participants was higher than that of the general population. According to a population census conducted in 2010 in Ehime Prefecture (Statistics Bureau Ministry of Internal Affairs and Communications 2012 2010), the proportions of persons aged 60–69 years with low, medium, high, and unknown educational levels were 28.2%, 48.6%, 19.0%, and 4.2%, respectively, in men, and 26.7%, 56.4%, 12.9%, and 4.0%, respectively, in women. The corresponding figures in the present study for persons aged 60–69 years were 11.8%, 55.3%, 32.9%, and 0.0%, respectively, in men, and 16.3%, 52.4%, 31.3%, and 0.0%, respectively, in women. On the other hand, the distributions of all three SNPs under study were within Hardy–Weinberg equilibrium, and any selection bias in relation to genotype distribution was negligible.

A score higher than 15 on the CES-D is considered to indicate suspected MDD (Li and Hicks 2010; Radloff 1977), but not all cases are diagnosed as MDD because an actual diagnosis of MDD should be made by psychiatrists. Moreover, the cutoff score of 16 on the CES-D may be inadequate for certain sex and racial/ethnic groups to evaluate depressive symptoms (Henry et al. 2018). The possibility of non-differential outcome misclassification would introduce a bias toward the null. The number of study subjects was rather small for a valid genetic association study; however, significant associations were detected. Although adjustment was made for some confounders, residual confounding effects could not be ruled out.

## Conclusions

The present case–control study showed that the GA and AA genotypes of *AGXT2* SNP rs180749 and the TAC haplotype of rs37370, rs180749, and rs16899974 were significantly associated with an increased risk of depressive symptoms based on the CES-D, whereas the TGC haplotype of these three SNPs was significantly related to a decreased risk of depressive symptoms. These findings should be confirmed by further epidemiological studies with larger numbers of participants. To clarify the relationship between *AGXT2* and depressive symptoms, further studies of the molecular biological mechanisms are also desirable.

## Data Availability

The data supporting this research cannot be made available for privacy or other reasons.

## Abbreviations

ADMA: Asymmetric *N*^G^, *N*^G^-dimethyl-l-arginine
SDMA: Symmetric *N*^G^, *N*′^G^-dimethyl-l-arginine
MDD: Major depressive disorder
AGXT2: Alanine-glyoxylate aminotransferase 2
SNP: Single nucleotide polymorphism
AICOS: Aidai Cohort Study
BMI: Body mass index
OR: Odds ratio
CI: Confidence interval
CES-D: Center for Epidemiologic Studies Depression Scale
